# A Predictive Model for Toxicity Effects Assessment of Biotransformed Hepatic Drugs Using Iterative Sampling Method

**DOI:** 10.1038/srep38660

**Published:** 2016-12-09

**Authors:** Alaa Tharwat, Yasmine S. Moemen, Aboul Ella Hassanien

**Affiliations:** 1 Faculty of Engineering, Suez Canal University, Egypt; 2 Scientific Research Group in Egypt, (SRGE), Cairo, Egypt; 3 Clinical Pathology Department, National Liver Institute, Menoufia University, Egypt; 4 Faculty of Computers and Information, Cairo University, Egypt

## Abstract

Measuring toxicity is one of the main steps in drug development. Hence, there is a high demand for computational models to predict the toxicity effects of the potential drugs. In this study, we used a dataset, which consists of four toxicity effects:mutagenic, tumorigenic, irritant and reproductive effects. The proposed model consists of three phases. In the first phase, rough set-based methods are used to select the most discriminative features for reducing the classification time and improving the classification performance. Due to the imbalanced class distribution, in the second phase, different sampling methods such as Random Under-Sampling, Random Over-Sampling and Synthetic Minority Oversampling Technique are used to solve the problem of imbalanced datasets. ITerative Sampling (ITS) method is proposed to avoid the limitations of those methods. ITS method has two steps. The first step (sampling step) iteratively modifies the prior distribution of the minority and majority classes. In the second step, a data cleaning method is used to remove the overlapping that is produced from the first step. In the third phase, Bagging classifier is used to classify an unknown drug into toxic or non-toxic. The experimental results proved that the proposed model performed well in classifying the unknown samples according to all toxic effects in the imbalanced datasets.

The development of novel drugs is a complex and an expensive process, and it has several steps^1^. Measuring toxicity of the drugs’ components is one of these steps. This step is very important as it is used to predict drug failures before any clinical trials. Hence, this step could save $100 million per one drug development in the US as reported in Food and Drug Administration (FDA)^2^,^3^. This reflects the importance of determining the toxicological effects as early as possible. For all of these reasons, toxicity measures for thousands of compounds become a hot topic in recent studies^4^,^5^.

Toxicity of a substance refers to the undesirable effect of the drug on the whole organism (e.g. animal), an organ (e.g. liver), or substructure of the organism (e.g. a cell). However, reliable high-throughput assays are expensive; hence, there is a high demand for computational models. Computational models offer a fast and cheap alternative to *in-vivo* and *in-vitro* bioassays. Moreover, the computational model protects animals and saves experimental materials. Thereby, using machine learning or fully automated system enables the pharmaceutical industry to produce over 100,000 new compounds yearly^6^,^7^.

Computational models are used to estimate toxicity for long ago. The aim of such models is to classify the toxicity of chemical compounds, the toxicological endpoints or the effect of different concentrations of the chemical compounds accurately. Pugazhenthi and Rajagopalan reported that machine learning is increasingly used in the pharmaceuticals’ research and development, especially, Particle Swarm Optimization, Support Vector Machines and Genetic Programming, which are suitable for noisy and high-dimensional data^8^. There are many examples of available computer models predicting toxicity such as Case^9^, TOPKAT^10^, OnkoLogic^11^, DEREK^11^ and Multicase^12^. To date, there is a large number of computational toxicity models have been developed, and increasing numbers of papers have been published^13^,^14^.

In this paper, a machine learning model was proposed to automatically evaluate the toxicity of chemical compounds. The toxicity risks of the current drugs include mutagenic effect, tumorigenic effect, irritant effect and reproductive effect. The current dataset is imbalanced, i.e. the samples of one class (positive class) significantly outnumber the samples of the other one (negative class). The proposed model consists of three phases. In the first phase, i.e. feature selection phase, the most discriminative features are selected using rough set-based methods. The data are pre-processed in the second phase, i.e. pre-processing phase, to obtain more balanced samples in each class. The selected features and the dataset that was pre-processed were then used to train the Bagging classifier in the third phase, i.e. classification phase. The Bagging classifier was then used to classify an unknown drug into toxic, i.e. has one of the toxic effects, or non-toxic.

## Description of the dataset

The current dataset is a benchmark of DataWarrior package^15^, and it was extracted from the Drug Bank database^16^. This dataset contained 6712 drugs, and these drugs were classified as follows: 1448 FDA-approved small molecule drugs, 131 FDA-approved biotech (protein/peptide) drugs, 85 nutraceuticals and 5080 experimental drugs. We used the drugs that were biotransformed in liver which estimated as 553 drugs^15^. Each drug is represented by 31 features or attributes, which were calculated using DataWarrior package^15^. These features are listed in Table 1. The current dataset includes four different toxic effects as depicted in Table 2, where the imbalance ratio is the number of samples of the majority class per each sample of the minority class. As shown in Table 2, the mutagenic, tumorigenic and irritant effects have high imbalance ratio, while the reproductive effect has low imbalance ratio. Moreover, the positive class represents the minor class, which may have a negative impact on the sensitivity of the proposed model. In this research, we considered each toxic effect as a separate dataset.

In Table 2, the reproductive effect considered the top risk effect (33.82%); mutagenic and tumorigenic effects are equal to (16.28%), finally irritant effect with (12.16%) for the current FDA drugs, which reflects burden on liver and such drugs should be replaced with more safe medications.

## Theory and Method

### Feature Selection using Rough Set Theory

The Rough set theory is a new mathematical approach to imprecision, vagueness and uncertainty^17^. In an information system, the data can be represented as a table. Each row of this table represents one object, and each column is one feature or attribute. Mathematically, an information system is denoted by *I* = (*U, A, V, f* ), where *U* represents a non-empty finite set of objects, i.e. the universe, *A* represents a non-empty finite set of features, *V* is the union of features domain as follows, *V* = *U*_*a*∈*A*_*V*_*a*_, and *f*_*a*_ : *U* → *V*_*a*_, where *V*_*a*_ is the set of values of feature *a*^18^,^19^,^20^,^21^. A Decision System has the same structure of data, but each object has its own decision, target or class label. For example, in our toxicity dataset, each object is represented by a set of features and a decision of that object, whether this object is toxic or not. Mathematically, a decision system *D* = (*U, A* ∪ *d, V, f* ), where *A* is the condition features and *d* represents a decision feature^19^.

Each non-empty subset *B* ⊆ *A* determines an equivalence relation as follows, *IND(B*) = {(*x, y*) ∈ *U* × *U*| ∀*a* ∈ *B, f*_*a*_(*x*) = *f*_*a*_(*y*)}. If (*x, y*) ∈ *IND(B*), then *x* and *y* are indiscernible by attributes from *B*^19^,^21^.

Given a subset *X* ⊆ *U* and a relation *H*, the lower approximation 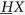 is defined as follows, 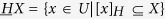, and the upper approximation 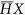 of *X* can be defined as follows, 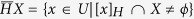.

Let *P, Q* ⊆ *A* be an equivalent relation over *U*, the positive, negative and boundary regions are defined as follows, 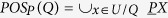, 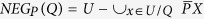, and 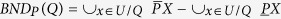, where *POS*_*P*_ (*Q*) is the positive region of the relation *U/Q* with respect to *P*, which represents the set of all objects of *U* that can be uniquely classified to blocks or classes of *U/Q*, by means of *P, NEG*_*P*_ (*Q*) is the negative region, and *BND*_*P*_ (*Q*) is the boundary region. The set is called rough or imprecise if it has a non-empty boundary region^19^,^21^.

Measuring dependency between attributes is an important task of data analysis. Given *P, Q* ⊆ *A*, and all features from the relation *P* are determined by the features from *Q*. If there is a relation between *P* and *Q*, then *P* depends totally on *Q (IND(P*) ⊆ *IND(Q*)) is denoted by *Q* ⇒ *P*, i.e. the partition that is generated by *P* is better than the partition generated by *Q*. The degree of dependency *k* is denoted by 
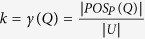
, where |*S*| represents the cardinality of *S*. If *k* = 1, then *P* depends totally on *Q*, on the other hand, if *k* = 0, then *P* does not depend on *Q*, if 0 < *k* < 1, then *P* depends partially on *Q*. In the decision systems, the degree of dependency represents the quality of approximation of classification^19^,^21^.

The goal of the feature reduction technique is to remove the redundant features so that the reduced set can achieve the same performance of classification as the original features. The reduct can be defined as a minimal subset *R* of the original features *C* such that *γ*_*R*_(*D*) = *γ*_*C*_(*D*), where *R* is the minimal subset if *γ*_*R*−*a*_(*D*) ≠ *γ*_*R*_(*D*), ∀*a* ∈ *R*. This means that there are no features can be removed from *R* without affecting the dependency degree. A decision table may have one or more attribute reducts. The set of all reducts is defined as follows, 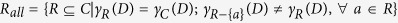. The rough set is used to find the reduct with the smallest cardinality which represents the global minimum. That is, an attempt to locate a single element of the reduct set (*R*_*min*_) as follows, *R*_*min*_ = {*R* ∈ *R*_*all*_, ∀ *Y* ∈ *R*_*all*_,|*R*| ≤ |*Y*|}^19^,^21^.

In this paper, three different rough set-based methods are used for feature selection; namely, Quick Reduct Feature Selection (QRFS)^20^,^22^, Discernibility Matrix-based Feature Selection (DMFS)^23^,^24^ and Entropy-Based Feature Selection (EBFS)^20^,^22^,^25^. Due to the paper length restrictions, we will not describe these algorithms here; more details can be found in the related references.

### Imbalanced datasets

The problem of imbalanced datasets appears frequently in the classification problem. The main property of this problem is that the samples of one class, i.e. majority class, significantly outnumber the samples of the other one, i.e. minority class^26^,^27^.

In binary classification, it is difficult for the classifier to learn from a minority class. This is because the data acquisition of the samples belongs to this class is expensive. Hence, most of the standard classifiers consider a balanced training data; but the minority samples are misclassified frequently. This is because the use of global assessment methods to evaluate the learning algorithm, such as accuracy rate, which might provide an advantage to the majority class^26^. There are many methods such as sampling methods^28^, Cost-Sensitive methods^29^ and Kernel-Based methods^27^ are used to solve the imbalanced dataset problem. In this paper, sampling methods are used to obtain more balanced samples in each class.

### Random Sampling

Sampling methods are widely used to solve the imbalanced datasets problem. These methods modify the prior distribution of the minority and majority classes in the training phase to obtain more balanced samples in each class. There are many sampling methods such as, *Random Under-sampling* (RUS), *Random Over-sampling* (ROS) and *Synthetic Minority Oversampling Technique* (SMOTE).

The aim of RUS method is to randomly extract a small set of the majority class to train the classifier while preserving all the minority samples. Hence, the training data become more balanced, and the training process becomes faster. This method is widely used when the number of samples of the majority class is very large. However, discarding samples may lead to neglect useful information and hence degrade the classifier performance^27^,^30^.

The goal of ROS method is to increase the size of the minority class by adding/replicating a set of samples that are sampled from the minority class. Hence, this method balances the class distribution through replicating the samples of the minority class; thus, improves the minority class recognition. The main disadvantage of this method is making exact copies of the existing samples which may lead to over-fitting. Another disadvantage of this method is increasing the number of training samples, which increases the learning time^27^,^30^.

In SMOTE method, the aim is to create data based on the similarities between existing minority samples. In other words, the minority class is over-sampled by creating synthetic samples. For each sample in the minority class *x*_*i*_ ∈ *S*_*min*_, the *k* nearest neighbors/samples are selected, where *S*_*min*_ is the set of minority class samples. A synthetic sample can be created as follow, 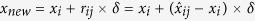, where *x*_*i*_ ∈ *S*_*min*_ is one of the minority class samples, 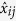 is one of the *k*-nearest neighbors for 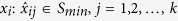, *k* is the number of selected neighbors, *δ* ∈ [0, 1] is a random number, and *x*_*new*_ is the new point/sample along the line joining *x*_*i*_ and 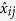 (see Supplementary Fig. S1). However, in SMOTE algorithm, the same number of the synthetic data are generated for each minority sample without consideration to neighboring samples, which may increase the overlapping between classes^27^,^31^,^32^. More details about SMOTE algorithm can be found in ref. 33.

### Assessment methods for imbalanced datasets

Accuracy is one of the most commonly used measures, and it is defined as a ratio between the correctly classified samples to the total number of samples as follows, 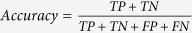, where *TP* is the true positive (number of correctly predicted toxic compounds), *FN* is the false negative (number of toxic compounds but not predicted to be toxic), *TN* represents the true negative (number of compounds that are correctly predicted to be not toxic) and *FP* is the false positive (the number of not toxic compounds, but predicted to be toxic). The accuracy does not distinguish between the numbers of corrected labels of different classes. Hence, in imbalanced datasets, the accuracy may lead to erroneous conclusions^34^.

Because of this, instead of using accuracy in imbalanced datasets, different assessment methods are considered. *Sensitivity* and *Specificity* are two appropriate metrics to measure the performance of classification over imbalanced datasets. Sensitivity, recall, or True Positive Rate (TPR) is defined as the ratio of true positive samples to the sum of true positive and false negative samples as follows, 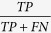. In the proposed model, sensitivity measures how well the toxicity model detects the toxic effect, i.e. positive cases. In other words, sensitivity represents the probability that the toxic case will be detected by the model as a positive, i.e. toxic case^35^. Specificity or True Negative Rate (TNR) is expressed as the ratio of the true negative samples to the sum of the true negative and the false positive samples as follows, 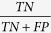. In the proposed model, specificity measures how well the toxicity model detects nontoxic cases, i.e. negative cases^35^. The main goal of all classifiers is to improve the sensitivity, without sacrificing the specificity. However, the sensitivity and specificity goals are often conflicting and attacking them simultaneously, which may not work well, especially when the dataset is imbalanced. Hence, Geometric Mean (GM) incorporates both sensitivity and specificity as in Equation (1). However, there are certain drawbacks associated with the use of *GM* to evaluate classifiers. For example, *GM* is ineffective to compare the performance of different classifiers over a range of sample distributions. For this reason, Receiver Operating Characteristic (ROC) assessment method makes use of the proportion of TPR and False Positive Rate (FPR), where FPR is the proportion of negative cases that were incorrectly classified as positive and it is calculated as follows, 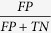 27,35,36. The ROC is a graphical approach for displaying the trade-off between TPR (the *X* axis), i.e. benefits that are reflected by true positives, and FPR (*Y* axis), i.e. costs that are reflected by false positives, of a classifier and any point in ROC curve represents the performance of a single classifier on a given distribution^27^,^37^.


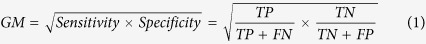


### Proposed Sampling Method: ITerative Sampling (ITS)

In order to overcome the drawbacks of random sampling, i.e. over-sampling, under-sampling and SMOTE algorithms, in this research, *ITerative Sampling* (ITS) method is proposed. This method is inspired from *k*-*Nearest Neighbor* classifier (*k*-NN)^38^. This method iteratively modifies the prior distribution of the minority and majority classes. In random sampling methods, the samples are removed or replicated randomly without consideration to neighboring samples, which may increase the overlapping between classes. On the other hand, the ITS method is proposed in this research to overcome this limitation. Iterative sampling method has two main steps. In the first step (*Sampling Step*), the data are iteratively under-sampled and over-sampled to increase the number of the minority class samples while simultaneously decreasing the majority class samples. In the second step, a *Tomek links* data cleaning technique is used to remove the overlapping that is may introduced from the first step.

In the first step, the data are iteratively under-sampled to reduce the number of majority class samples and then over-sampled to increase the number of minority class samples. The main difference between this method and standard random sampling methods, i.e. RUS and ROS methods, is that; (1) in RUS method, the samples are removed randomly, while in ITS method, the *Danger* samples are removed. The Danger samples represent the borderline majority class samples (the samples that are most likely to be misclassified) and the noisy samples, (2) in ROS method, the minority class samples that are not classified as danger samples are replicated (see Supplementary Fig. S2).

A brief description of the first step in ITS method is as follows (see Supplementary Fig. S3). In the under-sampling step, for each sample in the majority class (*x*_*i*_ ∈ *S*_*maj*_), a *k* nearest samples from *S* are selected (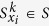). The sample *x*_*i*_ is removed if the minority class contains the most samples among 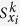, i.e. *x*_*i*_ is Danger. In the over-sampling step, for each sample in the minority class (*x*_*i*_ ∈ *S*_*min*_), a *k* nearest samples from *S* are selected (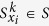). The sample *x*_*i*_ is replicated if the minority class contains the most samples among 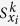, i.e. *x*_*i*_ is not Danger. This step will continue till the majority and minority classes are equal.

In the data cleaning step (see Supplementary Fig. S3), a Tomek links method is used to remove the overlapping between different classes due to the sampling step. Tomek link represents a pair of minimum distance nearest neighbors of different classes. Given two samples, *x*_*i*_ ∈ *S*_*min*_ and *x*_*j*_ ∈ *S*_*maj*_. The distance between *x*_*i*_ and *x*_*j*_ is denoted by *d(x*_*i*_, *x*_*j*_). The two samples (*x*_*i*_ and *x*_*j*_) is called a Tomek link if there is no sample *x*_*e*_, such that *d(x*_*i*_, *x*_*e*_) < *d(x*_*i*_, *x*_*j*_) or *d(x*_*j*_, *x*_*e*_) < *d(x*_*i*_, *x*_*j*_). All samples that represent Tomek links are removed until all closest neighbors pairs are from the same class (see Supplementary Fig. S2). Hence, this step is used to cleanup the unwanted overlapping between different classes after sampling step and hence the classification performance can be improved^27^,^39^.

### The Bagging Classifier

Ensemble classifier is a combination of multiple classifiers, referred as weak/single learners. A weak learner is a simple, fast and easy to implement classifier such as single level decision tree or simple neural networks. Ensemble classifiers usually achieve performance better than single classifiers^40^. There are many types of ensembles such as Bagging^41^, AdaBoost^42^ and Random Forest^43^. In this research, Bagging classifier is used.

**Figure 1:**
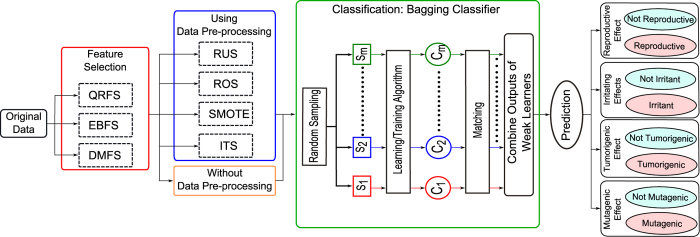
Block diagram of the proposed model.

Bagging classifier creates its ensemble by training different weak learners on a random distribution of a training dataset. The Bagging classifier consists of two phases, namely, training and testing phases. In the training phase, for each iteration (*t*), a number of training samples are selected randomly (*S*_*i*_) to train the current weak learner (*C*_*t*_) (see Fig. 1). Hence, in the resulting training set, many of the original samples may be repeated, while others may be left out. In the testing phase, an unknown sample (*x*_*test*_) is classified using all the weak learners that were trained in the training phase. The outputs of all weak learners are combined using majority voting method to determine the final decision (see Supplementary Fig. S4)^40^,^41^. More details about Bagging classifier can be found in ref. 41.

### Proposed Model

This section describes the proposed model in detail. The model, as illustrated in Fig. 1, generally consists of three phases: feature selection, data pre-processing and classification. In the first phase, rough set-based methods were used to select the most discriminative features. In this phase, a number of features were selected from the feature vector using three different rough set-based methods (QRFS, DMFS and EBFS). The aim of applying these algorithms is to reduce the number of features which reducing the classification time and improving the classification performance. In the second phase, different algorithms were used to obtain a balanced distribution of the classes. In other words, different sampling techniques were used to solve the problem of imbalanced datasets, where a novel algorithm, ITS and three well-known sampling methods, i.e. ROS, RUS and SMOTE, were used to solve this problem. In the last and third phase, the proposed model gives a decision about whether an input (i.e. unknown) drug sample is positive or negative. In this phase, Bagging classifier was used. As shown in Fig. 1, each weak learner of the Bagging classifier was used to classify the unknown sample and the outputs of all weak learners then combined to determine the final prediction. In this research, we consider each toxic effect as a separate dataset. For example, according to the mutagenic effect, the unknown sample is classified to be mutagenic or non-mutagenic (see Fig. 1). Similarly, each of the other three toxic effects is considered as a separate dataset.

## Results and Analysis

In this section, three experiments were conducted. The aim of the first experiment is to reduce the classification time by removing irrelevant features, in other words, select the most important features. The aim of the second experiment is to evaluate the proposed model using the original dataset, i.e. without pre-processing. The third experiment was conducted to demonstrate that the techniques for pre-processing dataset improved the classification performance for imbalance learning.

In all experiments, 5-fold cross-validation tests have used. In *k*-fold cross-validation, the original samples of the dataset was randomly partitioned into *k* subsets of (approximately) equal size and the experiment is run *k* times. For each time, one subset was used as the testing set and the other *k* − 1 subsets were used as the training set. Both training and testing samples were selected randomly.

### Feature selection experiment

The aim of this experiment is removing the irrelevant features to reduce the classification time and maintaining high accuracy, in the current work, some cases actually improved the accuracy. Three rough set-based methods, i.e. QRFS, EBFS and DMFS, were used to select the most discriminative features. The selected features are listed in Table 3.

From Table 3 many notices can be seen. Firstly, in most cases, the three feature selection methods achieved high reduction rate. Secondly, from the selected features, an important notice is that the intersection between the three feature selection methods represents the most important features in each toxic effect. In the table, the features that are highlighted in bold and underlined text characterize the most important features. For example, in mutagenic toxic effect, the most important features that were used to discriminate between mutagenic and non-mutagenic drugs were *H-Donors, Druglikeness, Molecular Shape Index, Molecular Flexibility* and *Rotatable Bonds*. Another important finding was that the twelfth and nineteenth features, i.e. *Molecular Flexibility* and *Rotatable Bonds*, were selected in all toxic effects, i.e. mutagenic, tumorigenic, reproductive and irritant, using all feature selection methods which reflects the importance of these two features.

**Figure 2:**
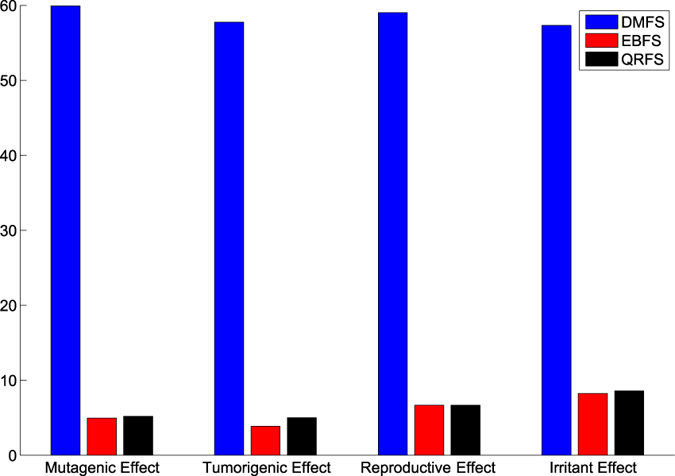
A comparison between QRFS, EBFS, and DMFS methods in terms of CPU time using mutagenic, tumorigenic, irritant, and reproductive effects.

In terms of computational time, the rough ordering of techniques was: EBFS < QRFS < DMFS as shown in Fig. 2. From this figure we note that the computational time of both EBFS and QRFS algorithms was much lower than DMFS algorithm. This is because the complexity of DMFS method is *O*((*N* + log*M)M*^2^), where *N* and *M* represent the number of features and objects, respectively. Hence, it needs a significant amount of time for the computation of the discernibility matrix, and the time was increasing quickly with increasing number of objects in the dataset^19^,^23^. On the other hand, the complexity of EBFS and QRFS is *O(N*^2^ + *N*)/2^22^. In this study, *N* = 31 and *M* = 553; thus, QRFS and EBFS need computational time lower than DMFS.

### Prediction of toxicity effects without using data pre-processing (original datasets)

**Figure 3:**
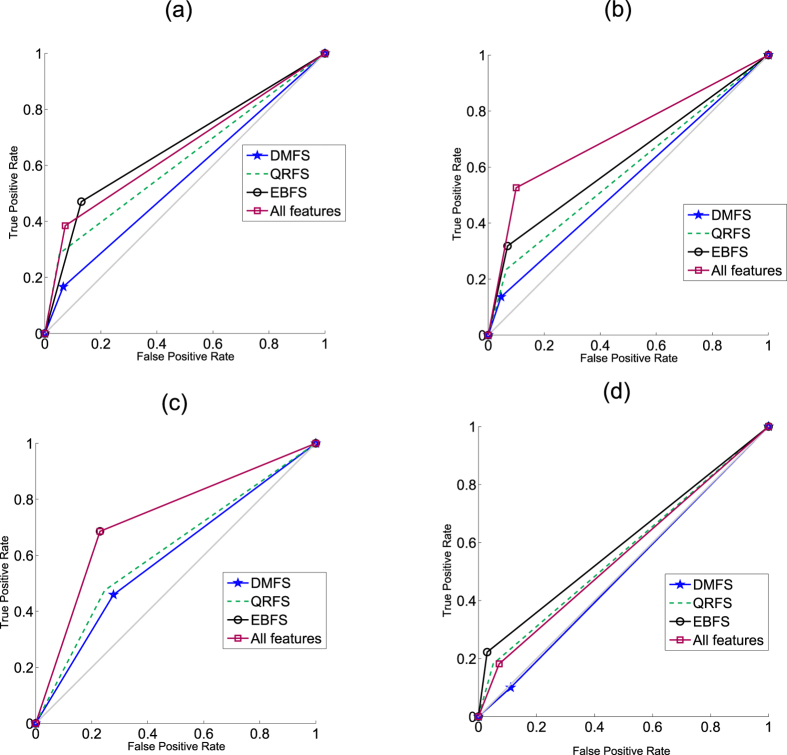
ROC curves of the proposed model using all and selected features: (**a**) Mutagenic effect, (**b**) Tumorigenic effect, (**c**) Reproductive effect and (**d**) Irritant effect.

**Figure 4:**
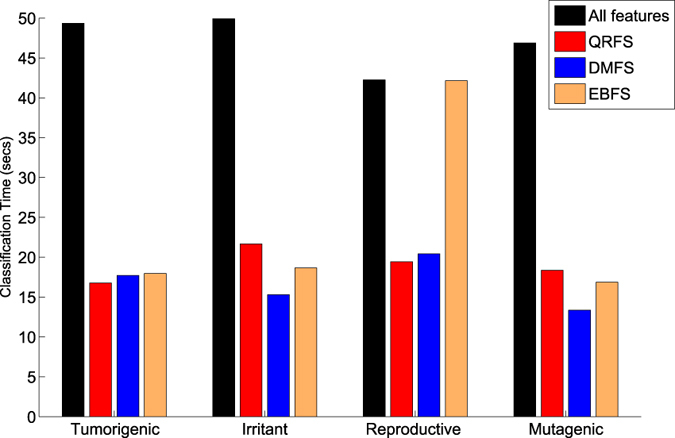
Classification time of the proposed model using the all and selected features.

The aim of this experiment is to evaluate the performance (i.e. accuracy, sensitivity, specificity and *GM*) of the proposed model when the original dataset, i.e. without pre-processing, was used. Moreover, the proposed model was tested when the whole (all) and selected features were used. In this experiment, Bagging classifier with three weak learners was used. The type of weak learner was the decision tree. The results of this experiment are summarized in Table 4. Moreover, Figs 3 and 4 show the ROC curves in addition to the classification time of the proposed model when the whole and selected features were used.

Table 4 compares the results of the proposed model when the original and selected features were used. From the table, many notices can be seen. First, the features that were selected using EBFS method achieved results better than the other methods. As shown, the EBFS method achieved the same results of all features when the reproductive dataset was used. Moreover, the EBFS method achieved the best results when the irritant dataset was used. All feature achieved the best results when the tumorigenic dataset was used, and the EBFS method achieved the second best results. In the mutagenic dataset, the EBFS achieved the best accuracy and sensitivity, while all features achieved the best specificity and GM. Second, in terms of sensitivity, the proposed model achieved low sensitivity compared with specificity. As shown, the sensitivity ranged from 20.4% to 55.8%. On the other hand, the proposed model achieved high specificity ranged from 75.3% to 89.1%. This means that the proposed model detects the nontoxic cases better than the toxic cases. It could be argued that the low sensitivity and high specificity were due to the imbalanced dataset problem. Further analysis showed that the proposed model achieved the lowest sensitivity (20.4% to 33.2%) when the irritant effect, which has the highest imbalance ratio was tested. On the other hand, the reproductive effect, which has the lowest imbalance ratio achieved high sensitivity (52.3% to 55.8%). Third, in terms of geometric mean, the EBFS method achieved the best results among all the other methods. Fourth, from the ROC curves in Fig. 3, it can be seen that the EBFS method achieved the best results, and these results are in agreement with GM results in Table 4. As shown in Fig. 3, EBFS achieved the best results when mutagenic and irritant datasets was used, while all features achieved the best results when the tumorigenic dataset was used. Generally, all features and EBFS method achieved results better than the other two methods.

To conclude, the selected features achieved good results compared with all features and the classification time was decreased as shown in Fig. 4. This figure shows that the classification time decreased when the number of features reduced. Moreover, the EBFS method achieved the best results. This is because: (1) In DMFS method, the attribute reducts represent the set of prime implicants that are reduced from the discernibility function^23^. However, as reported in refs 23 and 44, the simplification method for the discernibility matrix is not efficient, which may lead to a lower classification performance; (2) The ranking features in QRFS method depends on the dependency degree measure, while in EBFS method, the entropy measure was used for ranking features. However, the dependency measure depends mainly on the distribution of the dataset; on the other hand, the entropy-based methods are non-parametric; hence, the entropy measure became a well-used measurement in coding theory, communications engineering, and even the physical and biological sciences. For this reason, the EBFS method achieved results better than QRFS method, and these results are in agreement with those in ref. 45. Another important finding is that the sensitivity and GM of the proposed model were low due to the imbalanced datasets. The third experimental scenario was designed based on the results of this experiment to improve the sensitivity of the proposed model by (1) increasing the number of minority class samples or; (2) reducing the number of majority class samples.

### Prediction of toxicity effects using data pre-processing

**Figure 5:**
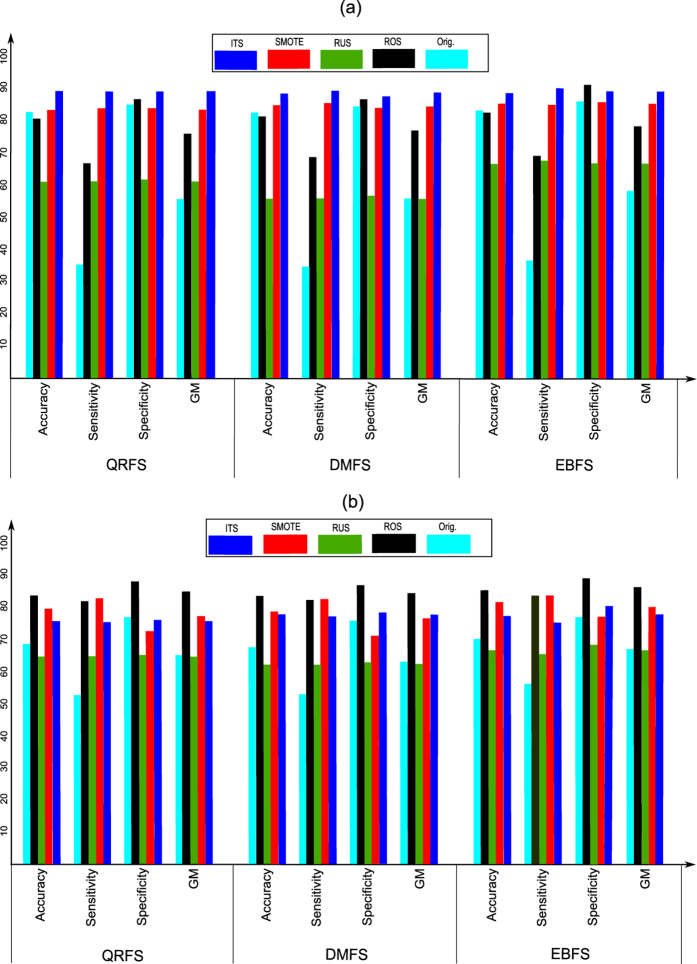
Results of classification tumorigenic and reproductive effects with and without pre-processing using the selected features from EBFS, QRFS and DMFS methods: (**a**) Tumorigenic effect, (**b**) Reproductive effect.

**Figure 6:**
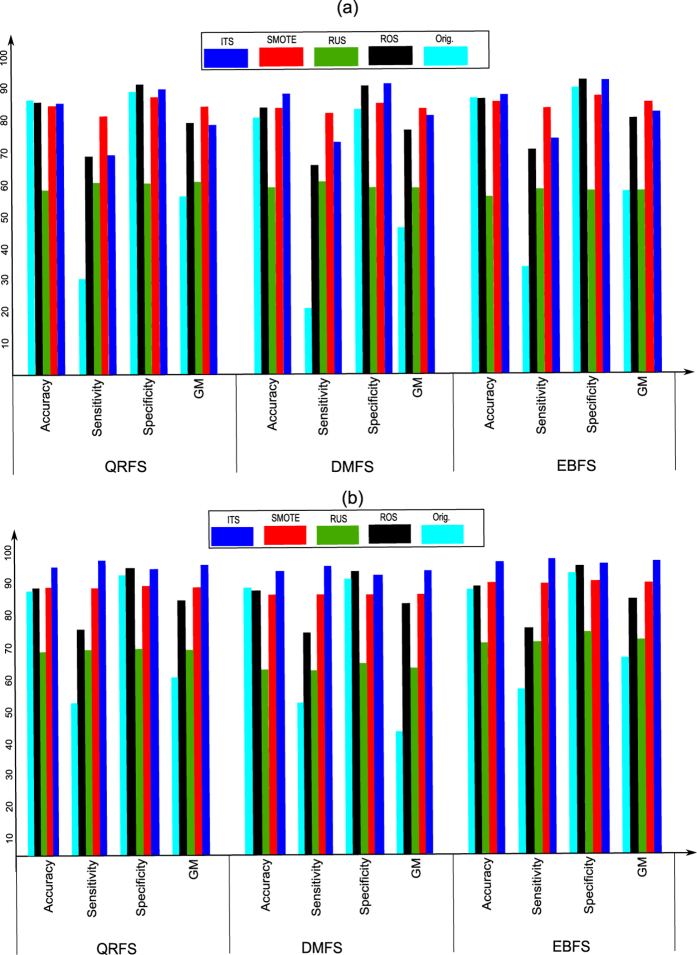
Results of classification irritant and mutagenic effects with and without pre-processing using the selected features from EBFS, QRFS and DMFS methods: (**a**) Irritant effect, (**b**) Mutagenic effect.

**Figure 7:**
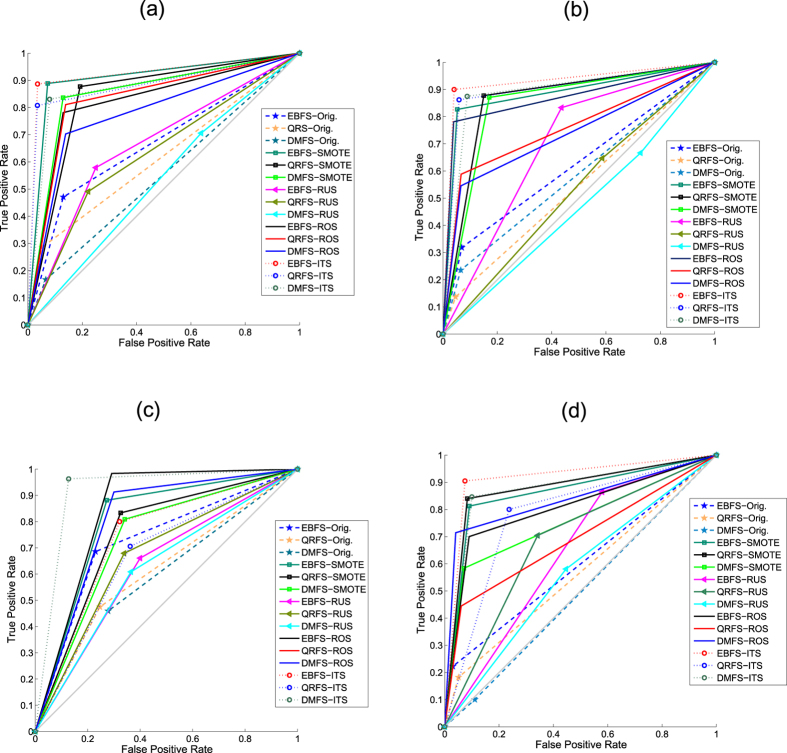
ROC curves of the proposed model using the original and selected features: (**a**) Mutagenic effect, (**b**) Tumorigenic effect, (**c**) Reproductive effect and (**d**) Irritant effect.

The aim of this experiment is to test the performance of the proposed model when different sampling methods were used to obtain balanced datasets. This experiment was divided into two sub-experiments. In the first sub-experiment, RUS, ROS and SMOTE algorithms were used to pre-process the datasets to obtain a balanced distribution of classes. In the ROS algorithm, the minority class was randomly over-sampled until the number of minority class samples matched the number of majority class samples. In the RUS algorithm, the majority class samples were randomly under-sampled until their number matched the number of minority class samples. In the SMOTE algorithm, the number of synthetic samples is a parameter in SMOTE algorithm. In this experiment, samples of minority class were synthesized to equalize the two classes. In this experiment, only the features that were selected using the three rough set methods were used. Moreover, in the Bagging classifier, only three weak learners were used and the type of weak learner was the decision tree. Figures 5 and 6 summarize the results of this experiment. Moreover, the results of the proposed model using the original dataset (Orig.), i.e. the dataset without pre-processing, are also summarized in the same figures. In addition, Fig. 7 compares the ROC curves of the proposed model when (1) the datasets was pre-processed using RUS, ROS and SMOTE algorithms; (2) the original dataset, i.e. without pre-processing, was used.

From Figs 5, 6 and 7 many notices can be seen as follows:

- **RUS**: As shown from figures, the accuracy of RUS algorithm was lower than the original dataset (55.7% to 66.5%). On the other hand, the RUS algorithm increased the sensitivity of the proposed model compared with the original dataset. As shown, the sensitivity ranged from 55.8% to 67.5%. Because the sensitivity and specificity goals are inversely proportional, the RUS algorithm achieved lower specificity. As shown, the specificity ranged from 56.6% to 69.3%. The reason for increasing sensitivity and decreasing specificity was due to the samples that were removed from the majority class. In terms of GM, the RUS algorithm achieved results better than the original dataset and these results are in agreement with ROC curves in Fig. 7. Moreover, in Fig. 7, EBFS method achieved the best results when the mutagenic and tumorigenic datasets was used, also the second best results when reproductive and irritant datasets was used.
- The results of RUS algorithm indicate that the RUS algorithm increased the sensitivity and decreased the specificity of the proposed model. Hence, RUS algorithm helps the proposed model to detect the positive cases better than the negative cases.
- **ROS**: It can be seen from the figures that the accuracy (80.3% to 85.8%), sensitivity (65% to 83.2%), specificity (86.3% to 91.7%) and GM (75.7% to 85.8%) of the ROS algorithm were better than RUS and the original dataset. In other words, the ROS algorithm achieved sensitivity and specificity higher than RUS and the original dataset. This is because there are no samples were removed in the ROS algorithm and the samples of minority class were over-sampled. Moreover, as shown in Fig. 7, the EBFS method achieved the best results when the tumorigenic and reproductive datasets was used, besides the second best results when the mutagenic and irritant datasets was used.
- **SMOTE**: From Figs 5, 6 and 7 it can be seen that the SMOTE algorithm improved the sensitivity of the proposed model. As shown, the sensitivity of the proposed model ranged from 80.6% to 84.6% when the SMOTE algorithm was used. Hence, the SMOTE algorithm achieved sensitivity better than RUS, ROS and original dataset. Moreover, in terms of GM, the SMOTE algorithm also achieved the best results (76.1% to 85.2%). In terms of specificity (70.8% to 86.6%), ROS algorithm and the original dataset achieved results better than SMOTE algorithm. Low specificity rates reduced the whole accuracy and hence the accuracy of ROS and the original dataset was also better than SMOTE. In addition, as shown in Fig. 7, the EBFS method achieved the best results when the tumorigenic, mutagenic and reproductive datasets was used, and the second best results when the irritant dataset was used.

The findings of this sub-experiment indicate that the SMOTE algorithm achieved the best results by increasing the sensitivity of the proposed model. This is because (1) the RUS algorithm had removed samples from the majority class; (2) in ROS algorithm, the replication of the minority class samples do not cause its decision boundary to spread or extend into the majority class region. Moreover, ROS algorithm improved the performance of the proposed model compared with the original dataset. On the other hand, RUS algorithm achieved the worst results because of the samples that were removed from the majority class. In addition, the best results of the proposed model achieved when the features that were selected by EBFS method were used.

In the second sub-experiment, the proposed sampling method, i.e. ITS algorithm, was used to pre-process the datasets to obtain a balanced distribution of classes. In ITS algorithm, the value of *k* was initialized with 15. In this sub-experiment, only the features that were selected using the three rough set methods were used. In addition, in the Bagging classifier, only three weak learners were used and the type of weak learner was the decision tree. The results of this sub-experiment are illustrated in Figs 5 and 6. Moreover, the ROC curves of this sub-experiment are illustrated in Fig. 7.

From Figs 5, 6 and 7 many notices can be seen. First, the proposed sampling method achieved accuracy higher than all the other methods in most cases. Second, the sensitivity (68.4% to 92.2%) and GM (74.9% to 91.3%) of ITS method were better than all other methods when classifying mutagenicity and tumorigenicity effects, while the results of SMOTE method were better than all the other algorithms when classifying the irritant and reproductive effects. Third, the ITS algorithm achieved specificity (75.7% to 91.4%) rates higher than all the other algorithms. Figure 7 shows that the ITS method using EBFS feature selection method achieved the best results when the tumorigenic, mutagenic and irritant datasets were used, also the ITS using DMFS method achieved the best results when the reproductive dataset was used.

**Figure 8:**
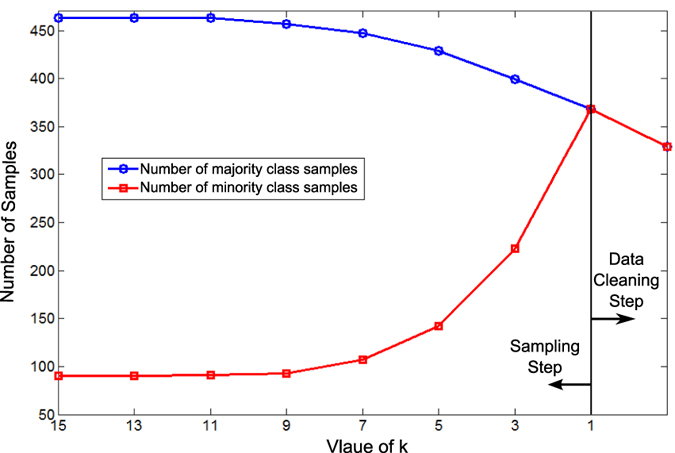
The number of samples in minority and majority classes in the two steps of ITS method using mutagenic effect dataset and the selected features by EBFS algorithm.

Figure 8 shows the number of minority and majority samples in the two steps of the ITS algorithm using mutagenic effect dataset and the features that were selected using EBFS method. In Fig. 8, the majority and minority classes have 463 and 90 samples, respectively. In the first step, i.e. sampling step, the majority class samples were under-sampled, while the minority class samples were over-sampled iteratively. The two classes have the same number of samples when *k* = 1. In the data cleaning step, 39 samples from each class which represent Tomek link were removed.

**Figure 9:**
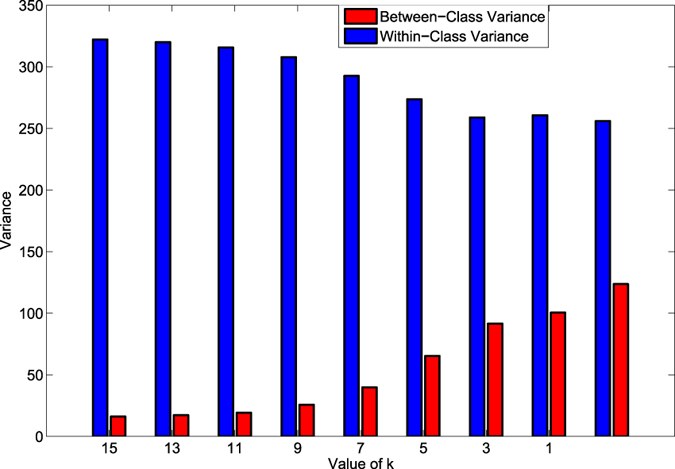
The between-class and within-class variance of the minority and majority classes in the two steps of ITS method using mutagenic effect dataset and the selected features by EBFS algorithm.

Figure 9 shows the between-class variance and total within-class variance of the ITS algorithm using mutagenic effect dataset and the features that were selected using EBFS method. The between-class variance represents the variance between positive and negative classes in our problem, in other words, the distance between the two classes, while the total within-class variance is the total of the within-class variance of the two classes. As shown, the between-class variance increased as the iterations proceeded in the sampling step. Moreover, the between-class variance increased also in the data cleaning step. On the contrary, the within-class variance decreased as the iterations proceeded in the sampling step, and also it was decreased in the data cleaning step. From these two findings, we can conclude that the ITS method improved the classification performance by increasing the distance between different classes and making the samples of each class closer to the mean of that class.

The findings of the second sub-experiment indicate that the proposed sampling algorithm achieved the best results when classifying mutagenic and tumorigenic effects, besides achieving competitive results when classifying irritant and reproductive effects. This is because; (1) RUS algorithm removes samples randomly, while the ITS algorithm removes the Danger samples; (2) ROS and SMOTE algorithms, respectively, replicate or generate samples randomly, while in the ITS algorithm, only the non-Danger samples are replicated; (3) data clean step in the ITS algorithm removes the overlapping between classes that is produced from the sampling step and hence increases the classification performance.

## Conclusions

A limitation that decreases the reliability of all toxicity prediction models is that experimentally determined toxic data is available for only a very small portion of compounds, compared with the actual number of toxic compounds. Another important limitation is the imbalanced datasets, due to the small number of positive samples. These limitations have a negative impact on the sensitivity of prediction models.

In this research, the toxic effects (risk factors) of the current drugs (liver biotransformation of drugs) are assessed. Four toxic effects (mutagenic effect, tumorigenic effect, irritant effect and reproductive effect) are assessed in this paper. The proposed model consists of three phases. In the first phase, the most discriminative features that were used to separate between positive and negative classes were selected using rough set-based methods. In the second phase, the proposed model was evaluated when the dataset was pre-processed in the framework of imbalanced datasets. In our experiments, RUS, ROS and SMOTE sampling methods were compared with the proposed sampling method, i.e. ITS. The results showed that all sampling methods that were used to address the imbalanced problem improved the overall classification performance of all toxic effects. Moreover, the proposed sampling method achieved the best results and developed the sensitivity of the proposed model. The Bagging classifier was used in the third phase to classify an unknown drug according to all toxic effects. The experimental results showed that the proposed model performed well in classifying the unknown samples according to all toxic effects in the imbalanced datasets. The sensitivity value of 92% means that a drug sample predicted as toxic, e.g. mutagenic, has a high probability to be mutagenic in reality.

## Additional Information

**How to cite this article**: Tharwat, A. *et al*. A Predictive Model for Toxicity Effects Assessment of Biotransformed Hepatic Drugs Using Iterative Sampling Method. *Sci. Rep.* **6**, 38660; doi: 10.1038/srep38660 (2016).

**Publisher's note:** Springer Nature remains neutral with regard to jurisdictional claims in published maps and institutional affiliations.

## Supplementary Material

Supplementary Materials

## Figures and Tables

**Table 1:** Dataset description.

Feature No.	Name	Feature No.	Name
1	Total Molecular Weight	17	Electron Negative Atoms
2	Molecular Weight	18	Stereo Centers
3	Absolute Weight	19	Rotatable Bonds
4	cLogP (Octanol/Water, partition coefficient)	20	Rings
5	cLogS (Aqueous solubility)	21	Aromatic Rings
6	H-Acceptors (Hydrogen bond Acceptor)	22	Aromatic Atoms
7	H-Donors (Hydrogen bond donor)	23	sp3-Atoms
8	Total Surface Area	24	Symmetric atoms
9	Polar Surface Area	25	Amides (acid amide)
10	Druglikeness	26	Amines
11	Molecular Shape Index	27	AlkylAmines
12	Molecular Flexibility	28	Aromatic Amines
13	Molecular Complexity	29	Aromatic Nitrogen
14	Non Hydrogen Atoms	30	Basic Nitrogen
15	Non-Carbon/Hydrogen Atoms	31	Acidic Oxygen
16	Metal Atoms		

**Table 2:** Distribution of the two classes of each toxic effect.

Toxic effect	#Samples in Positive Class	#Samples in Negative Class	Imbalance ratio
Mutagenic Effect	90 = 16.28%	463 = 83.73%	5.14
Tumorigenic Effect	90 = 16.28%	463 = 83.73%	5.14
Reproductive Effect	187 = 33.82%	366 = 66.18	1.96
Irritant Effect	67 = 12.16%	486 = 87.88%	7.25

**Table 3:** The selected features using QRFS, DMFS and EBFS rough set methods.

Rough Set reduction method	Mutagenic Effect		Tumorigenic Effect		Irritant Effect		Reproductive Effect	
	Selected features	No. of features (Red. Rate %)	Feature Subset	No. of features (Red. Rate %)	Selected features	No. of features (Red. Rate %)	Selected features	No. of features (Red. Rate %)
QRFS	{1, 4, **7**, 8, **10**, **11**, **12**, 18, **19**, 20, 24, 25, 30}	13 (≈58.1%)	{2, **4**, **5**, **10**, 11, **12**, **13**, **18**, **19**, **24**, 27}	11 (≈64.5%)	{4, **5**, 6, **7**, **8**, 10, **11**, **12**, **18**, **19**, **20**, **24**, 25, 29}	14 (≈54.8%)	{**1**, 2, **4**, 8, **10**, **11**, **12**, **13**, **19**, **22**, **24**, 25, 26, 29}	14 (≈54.8%)
DMFS	{4, 5, **7**, **10**, **11**, **12**, 13, **19**, 22}	9 (≈71%)	{**4**, **5**, **10**, 11, **12**, **13,** **18**, **19**, 20, 22, **24**}	11 (≈64.5%)	{**5**, **7**, **8**, **11**, **12**, 13, **18**, **19**, **20**, 22, **24**}	11 (≈64.5%)	{**1**, **4**, 5, 7, **10**, **11**, **12**, **13**, 18, **19**, 20, **22**, 23, **24**}	14 (≈54.8%)
EBFS	{5, 6, **7**, **10**, **11**, **12**, 14, **19**, 22, 24, 30}	11 (≈64.5%)	{**4**, **5**, 6, 8, 9, **10**, **12**, **13**, **18**, **19**, **24**, 30}	12 (≈61.3%)	{4, **5**, **7**, **8**, 10, **11**, **12**, 13, **18**, **19**, **20**, **24**, 30}	13 (≈58.1%)	All Features	31 (0%)

**Table 4:** Accuracy, sensitivity, specificity and geometric mean (GM) of the proposed model using all features and the selected features using QRFS, DMFS and EBFS rough set methods.

Assessment Method	Mutagenic Effect				Tumorigenic Effect				Irritant Effect				Reproductive Effect			
	All	QRFS	DMFS	EBFS	All	QRFS	DMFS	EBFS	All	QRFS	DMFS	EBFS	All	QRFS	DMFS	EBFS
Accuracy	82.6	82.5	81.4	**82.9**	**84.2**	82.5	82.3	83	85	85.8	80.0	**86.1**	**69.8**	68.3	67.2	**69.8**
Sensitivity	49.8	46.7	47.4	**51.6**	**38.3**	35.2	34.7	36.5	27.2	29.9	20.4	**33.2**	**55.8**	52.3	52.6	**55.8**
Specificity	**88.2**	87.4	86.9	87.9	**86.4**	84.9	84.3	85.9	89	88.3	82.6	**89.1**	**76.7**	76.5	75.3	**76.7**
GM	**61.2**	55.2	38.3	62.6	**60.2**	55.4	55.7	58.2	50.5	52.4	45.5	**56.8**	**66.8**	64.8	62.7	**66.8**
