# Supplementary material for: A Predictive Model for Toxicity Effects Assessment of Biotransformed Hepatic Drugs Using Iterative Sampling Method
Source: Sci Rep. 2016 Dec 9;6:38660. doi: 10.1038/srep38660 (PMC5146749; doi:10.1038/srep38660)
Supplement: Supplementary Materials [file srep38660-s1.pdf]

Supplementary Information for: A Predictive Model for Toxicity Effects Assessment of Biotransformed Hepatic Drugs Using Iterative Sampling Method

Alaa Tharwat[1,2,*], Yasmine S. Moemen[2,3], Aboul Ella Hassanien[2,4,*]

[1]*Faculty of Engineering, Suez Canal University, Egypt*
[2]*Scientific Research Group in Egypt, (SRGE), http://www.egyptscience.net*
[3]*Clinical Pathology Department, National Liver Institute, Menoufia University, Egypt*
[4]*Faculty of Computers and Information, Cairo University, Egypt*

Supplementary Figures

1. Fig. S1: An example of SMOTE method, (a) before applying SMOTE method and (b) after applying SMOTE method.
2. Fig. S2: An example of ITS method. (a) Original data; (b) After applying sampling step; (c) The identified Tomek Links, (d) The dataset after removing Tomek links.
3. Fig. S3: ITS algorithm.
4. Fig. S4: Bagging Classifier Algorithm.

1-Supplementary Figure S1

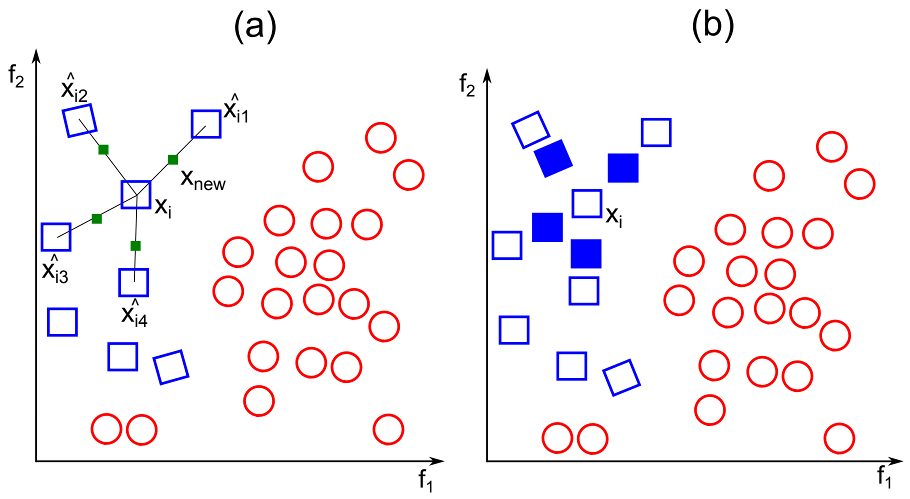

Fig. S1 An example of SMOTE method, (a) before applying SMOTE method and (b) after applying SMOTE method.

Correspondence to: <engalaatharwat@hotmail.com, aboitcairo@gmail.com>

Figure S1 illustrates an example of the SMOTE method. Figure S1(a) shows a typical imbalanced data distribution, where the circles and squares represent samples of the majority and minority classes, respectively. In this example, assume $k = 4$. Figure S1(a) shows the created samples along with the line segment between x_i and $\hat{x_{ij}}$. These samples are highlighted by the green square shape. These synthetic samples increase the number of minority samples and hence, significantly improves the performance of learning algorithm. Figure S1(b) shows the synthesized samples that are highlighted by solid squares. However, in SMOTE algorithm, the same number of the synthetic data are generated for each minority sample without consideration to neighboring samples, which may increase the overlapping between classes.

2-Supplementary Figure S2

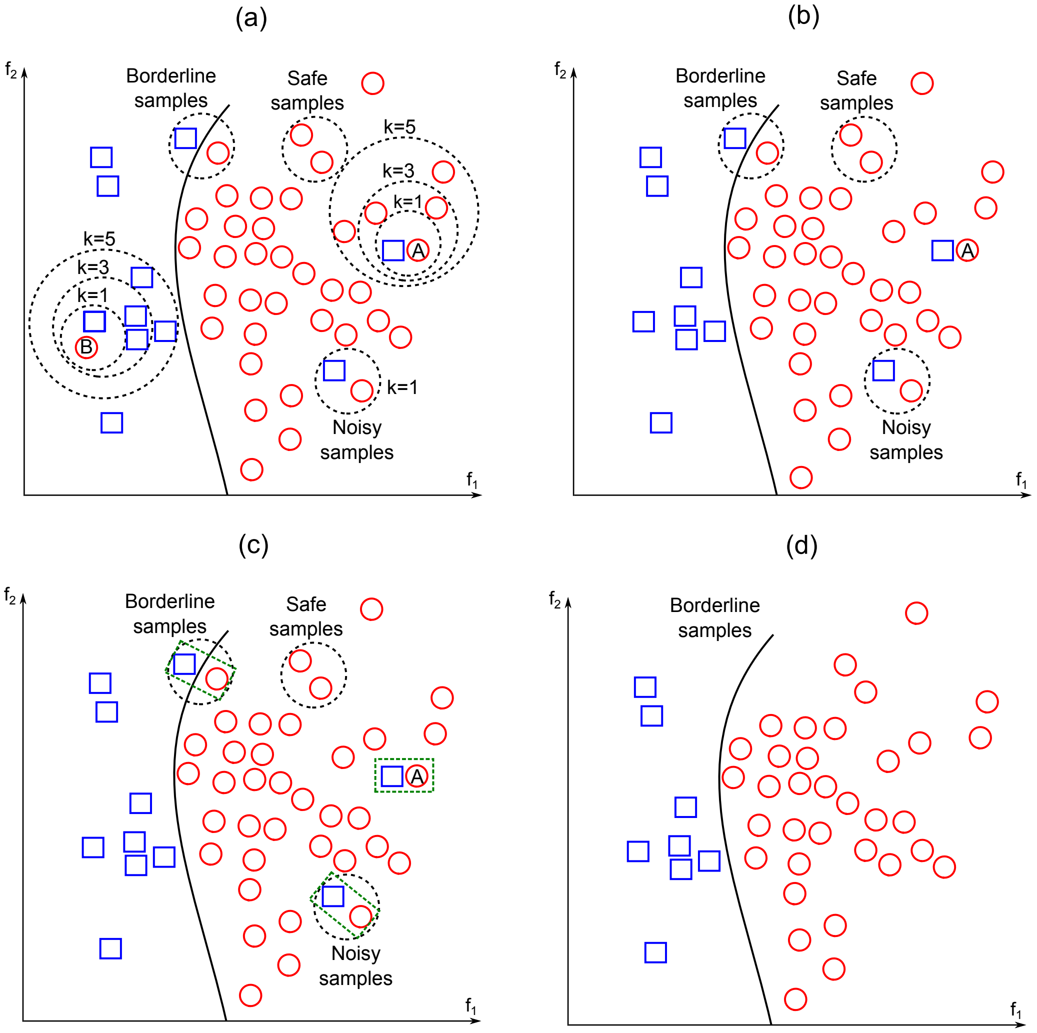

Fig. S2 An example of ITS method. (a) Original data; (b) After applying sampling step; (c) The identified Tomek Links, (d) The dataset after removing Tomek links.

Figure S2 illustrates an example of the ITS method. This figure shows the difference between borderline, safe and noisy samples (see Fig. S2 (a)). As shown in Fig.Fig. S2(a), the sample/point (A) when $k = 5$ or $k = 3$ is not classified as a danger sample, while the sample is classified as a danger sample when $k = 1$.

On the contrary, the sample B is classified as a danger sample when $k = 1, 3$, or 5. Hence, in the sampling step, when $k = 5$, the sample B is removed while sample A is preserved when the majority class samples are under-sampled as shown in Fig. S2(b). Similarly, the danger points in minority class will not be replicated. Figure S2(c) shows a data cleanup step. As shown in Fig. Fig. S2(c), there are three Tomek links are identified and represented by a green dashed box. Figure S2(d) shows the data after the data cleaning step, where the overlapping data samples are removed. As shown in Fig. S2(d), this step produces separated classes, which improves the classification performance.

3-Supplementary Figure S3

```
Input: Given a set of majority class samples S_maj, a set of minority class samples S_min, the number of nearest neighbors k=1,
n^- is the number of minority class samples and n^+ is the number of majority class samples.

Sampling Step
  [Under-Sampling]
  for k=K to 1
      for all Samples, x^i ∈ S_maj do
          Select the set of nearest k samples to x^i; call this set S^k_xi.
          if (the minority class contains the most samples among
           the selected samples S^k_xi (i.e. Danger sample)) then
              Remove x_i.
          end if
          if (n^-=n^+) then
              break.
          end if
      end for
  [Over-Sampling]
      for all Samples, x_i ∈ S_min do
          Select S^k_xi.
          if (the minority class contains the most samples among
           the selected samples S^k_xi (i.e. not aDanger sample)) then
              Replicate x_i.
          end if
          if (n^-=n^+)
              break;
          end if
      end for
      k=k-2.
  end for

Data Cleaning Step
  for i=1: n^-
      for j=1: n^+
          f=0 (where f is a boolean flag variable)
              for all Samples, x_k ∈ S_min, where k≠i do
                  if (d(x_k,x_i)≤d(x_i,x_j)) then
                      f=1 (i.e. This point is not a Tomek link)
                  end if
              end for
              for all Samples, x_k ∈ S_maj, where k≠j do
                  if (d(x_k,x_i)≤d(x_i,x_j)) then
                      f=1
                  end if
              end for
              if (f=0) then (i.e. This point is a Tomek link)
                Remove samples x_i and x_j
              end if
      end for
  end for
```

Fig. S3 ITS algorithm.

4-Supplementary Figure S4

Bagging Classifier Algorithm

Input: Given a training set $X=\{(x_1,y_1),.....,(x_M,y_M)\}$, where y_i is the class label of the sample x_i and M denotes the total number of samples in the training set.

Training Step

while ($t<Max_{iter}$), where Max_{iter} is the maximum number of iterations) **do**

Select a sample S_t from X.

Use S_t to train the current weak learner C_t.

end while

Testing Step

Given an unknown sample x_{test}.

Classify x_{test} using all weak learners (C_i, i=1,.... ,Max_{iter}).

Combine the outputs of all weak learners to determine the final prediction.

Fig. S4 Bagging Classifier Algorithm.
